# Improved Release of a Drug with Poor Water Solubility by Using Electrospun Water-Soluble Polymers as Carriers

**DOI:** 10.3390/pharmaceutics14010034

**Published:** 2021-12-24

**Authors:** Muriel Józó, Nóra Simon, Lan Yi, János Móczó, Béla Pukánszky

**Affiliations:** 1 Laboratory of Plastics and Rubber Technology, Department of Physical Chemistry and Materials Science, Budapest University of Technology and Economics, H-1521 Budapest, Hungary; jozo.muriel@vbk.bme.hu (M.J.); nora.smn@hotmail.com (N.S.); MinozBoGum@gmail.com (L.Y.); pukanszky.bela@vbk.bme.hu (B.P.); 2 Institute of Materials and Environmental Chemistry, Research Centre for Natural Sciences, ELKH Eötvös Loránd Research Network, H-1519 Budapest, Hungary

**Keywords:** valsartan, hydroxypropyl-methyl-cellulose, polyvinyl-pyrrolidone, polyvinyl-alcohol, drug morphology, distribution, pH, drug release

## Abstract

In an attempt to improve the solubility of valsartan, a BCS II drug, fibers containing the drug were prepared from three water-soluble polymers, hydroxypropyl-methyl-cellulose (HPMC), polyvinyl-pyrrolidone (PVP), and polyvinyl-alcohol (PVA). Fiber spinning technology was optimized for each polymer separately. The polymers contained 20 wt% of the active component. The drug was homogenously distributed within the fibers in the amorphous form. The presence of the drug interfered with the spinning process only slightly, the diameters of the fibers were in the same range as without the drug for the HPMC and the PVA fibers, while it doubled in PVP. The incorporation of the drug into the fibers increased its solubility in all cases compared to that of the neat drug. The solubility of the drug itself depends very much on pH and this sensitivity remained the same in the HPMC and PVP fibers; the release of the drug is dominated by the dissolution behavior of valsartan itself. On the other hand, solubility and the rate of release were practically independent of pH in the PVA fibers. The different behavior is explained by the rate of the dissolution of the respective polymer, which is larger for HPMC and PVP, and smaller for PVA than the dissolution rate of the drug. The larger extent of release compared to neat valsartan can be explained by the lack of crystallinity of the drug, its better dispersion, and the larger surface area of the fibers. Considering all facts, the preparation of electrospun devices from valsartan and water-soluble polymers is beneficial, and the use of PVA is more advantageous than that of the other two polymers.

## 1. Introduction

The most convenient method for the application of a drug is oral administration [1,2,3]. The approach has several advantages including the good cooperation of the patient, formulation freedom, and cost effectiveness. Consequently, pharmaceutical companies prefer the production of drugs for oral administration. However, in many cases, the physical-chemical characteristics of the drug do not favor oral administration because of either limited solubility or poor permeability [4,5,6,7,8,9]. The most important drawback of oral administration is small bioavailability and one of the basic conditions of large bioavailability is the sufficient solubility of the drug in water-based solution, like body fluids. The solubility of many drugs, especially those belonging to the Biopharmaceutics Classification System (BCS II) class, is small, thus various measures must be taken to increase it and improve bioavailability.

After the recognition of the importance of solubility in drug delivery, various physical and chemical approaches have been developed to improve it. Chemical methods, i.e., the modification of the chemical structure of the drug [10,11], are usually complicated and expensive, thus physical methods are preferred, if their use is possible. The most important physical methods are the decrease of particle size [3], the change of crystallization conditions [12], the preparation of solid dispersions [13,14,15], and solubilization [16,17]. The simplest of these approaches is to decrease the size of the particles by grinding or micronization [18,19]. Smaller particles have larger specific surface area thus the rate of dissolution increases. Active molecules are often solubilized by complexation, by the combination of the drug with a host molecule, the most frequently with cyclodextrins of surfactants [17,20,21]. Recently the innovative technique of using supercritical antisolvent coprecipitation was used to obtain nano-sized particles containing various active components [22,23].

Valsartan blocks selectively the angiotensin receptor thus hindering the coupling of angiotensin II to its receptor, which regulates blood pressure. Angiotensin II may also cause structural changes in the heart and the arteries. The bioavailability of valsartan is poor, two hours after the administration of a 80 mg dose, the concentration of the plasma is 1.64 mg/L [24]. Valsartan is a white, hygroscopic powder, which can be produced in both amorphous and crystalline form. Its crystal form may change and depends on the conditions of crystallization. The melting point of the crystals is usually between 50 and 110 °C. The drug is a tetrazole derivative containing carboxyl and carbonyl groups. It is practically insoluble in water but dissolves easily in methanol and ethanol. Its solubility depends strongly on the pH of the dissolution medium. The drug is produced as tablets, sometimes with auxiliary components (hydrochlorothiazide, amlodipine, lactose monohydrate, microcrystalline cellulose, etc.).

The importance of valsartan is shown by the numerous attempts to improve its solubility. Many of these approaches use spray drying in the presence of some additives serving as matrix. Beak et al. [25], for example, used Caproil 90, hydroxymethyl-cellulose, and various tensides and could produce homogeneous particles in the 100 nanometer range resulting in a considerable increase in bioavailability. Other groups [26,27] followed the same route of spray drying in combination with polymers and surfactants and invariably claimed the improvement of bioavailability. Capello et al. [28] on the other hand used cyclodextrin to increase the solubility of valsartan, while Youn et al. [29] attempted to reach the same goal by the recrystallization of the drug. Practically all approaches resulted in smaller particles and thus faster dissolution and better bioavailability.

A wide variety and devices are prepared to achieve the controlled delivery and increased release rate of the most diverse drugs and active components from nanoparticles [30,31] through membranes [32] to nanofibers [33,34,35,36]. These devices are fabricated from a wide range of materials including natural [36] and synthetic polymers [34], hydrogels [30,35,36] and composite materials [31,32,34]. Nanofibers, and specifically electrospun fibers, are often the preferred solution as shown also by the references cited above. According to the best of our knowledge, just several attempts have been made to use electrospinning for the improvement of the solubility of valsartan yet [37,38]. The method is used more and more frequently in medicine, because it has numerous advantages [39]. The spinning technology offers large flexibility in the production of the fibers and in formulation, and frequently offers the possibility to control release kinetics. The drug may be distributed in the polymer in amorphous form as small particles or even as dissolved molecules, and the small diameter of the fibers results in large contact surface accelerating release even further. Fiber characteristics depend on several factors including the characteristics of all components like the polymer, the solvent and the drug, as well as on processing parameters as voltage, pump rate, and the distance to the collector [40]. The release of the drug from the device is influenced by all these parameter, but also by the interaction of the components which may change the form and location of the drug in the fibers and thus release kinetics [41,42]. Although many parameters influence drug release, they also offer a possibility to regulate solubility and dissolution rate at the same time.

Taking into account all the considerations described above, the goal of our study was to improve the solubility of valsartan by incorporating it into electrospun water-soluble polymers. The polymers selected were hydroxypropyl-methyl-cellulose (HPMC), polyvinyl-pirrolidone (PVP), and polyvinyl-alcohol (PVA), all approved and used in pharmaceutics. The water solubility of the polymers allows rapid dissolution thus increasing release rate and bioavailability. Electrospinning conditions were optimized during the study and then drug release was determined as a function of the pH of the dissolution medium. Release rate was modeled using the Noyes–Whiney equation and the results were interpreted from the point of view of practical application.

## 2. Experimental

### 2.1. Materials

The valsartan used in the experiments was supplied by Egis Pharmaceutical PLC (Budapest, Hungary). Three water-soluble polymers were selected for the study. Hydroxypropyl-methyl-cellulose (Methocel E5, HPMC) was purchased from Colorcon Limited (Harleysville, PA, USA), polyvinyl-pirrolidon (PVP) was obtained from Alfa Aesar, (Tewksbury, MA, USA), and polyvinyl-alcohol (Mowiflex LP, PVA) from Kuraray (Tokyo, Japan). The chemicals used for the preparation of solutions and buffers, i.e., disodium hydrogenphosphate-2-hydrate, ethanol, methylene chloride, sodium hydroxide, and hydrochloric acid (37%) were purchased from Molar Chemicals (Budapest, Hungary), acetic acid from Merck (Darmstadt, Germany) and sodium acetate-3-hydrate from Reanal (Budapest, Hungary). All materials and chemicals were used as received.

### 2.2. Solutions

HPMC was dissolved in the 1:2 mass ratio mixture of ethanol and methylene chloride. A 15 wt% solution was prepared from 2 g polymer, which was diluted to 5, 7.5, and 10 wt% solution for electrospinning. Ethanol was used as solvent for the preparation of PVP solutions. The concentration of the polymer changed between 20 and 45 wt% during the optimization of the electrospinning technology. PVA was dissolved in distilled water to prepare solutions containing the polymer at 9, 11, 13, and 15 wt%. In order to improve the quality of the fibers and find optimum conditions, water was replaced with 10, 20, 30, and 40 vol% ethanol. HPMC and PVP solutions were prepared by continuous stirring overnight. PVA could be dissolved at 70–90 °C in 20–30 min by intensive stirring. The active component, valsartan, was dissolved in all three solution at 20 wt% related to the amount of the polymer.

Release experiments were carried out in four media, in buffers with three different pH values and in distilled water. The solutions were prepared in a calibrated measuring vessel of 1000 mL capacity. The composition of the buffer solution of 4.0 and 6.8 was taken from the literature [43]. The solution with pH 1.2 was prepared with 5.26 mL concentrated (37%) hydrochloric acid. The acetate buffer (pH 4.0) contained 4.7 mL concentrated (100%) acetic acid, 2.45 g C_2_H_3_O_2_Na·3H_2_O, and 2 mL 1 M NaOH solution. The phosphate buffer with pH 6.8 was produced from 6.12 g NaH_2_PO_4_, 8.72 g Na_2_HPO_4_·2H_2_O, and 2 mL 1 M NaOH solution. The pH of the solutions was checked with the help of a Metrohm 827 pH apparatus (Metrohm Ltd., Herisau, Switzerland) and was adjusted by adding the necessary amount of 1 M NaOH solution.

### 2.3. Electrospinning

Fibers were fabricated using the Spinsplit (Spinsplit LLC, Budapest, Hungary) electrospinning machine. Concentration, voltage, pump rate, and the distance to the collector plate all depend on the characteristics of the solutions, i.e., on the combination of the polymer and solvent. The optimized parameters were different for the three polymers used. The time of fiber spinning changed between 5 and 30 min depending on the amount of fiber needed.

### 2.4. Release Experiments

Release experiments were carried out on 6 mg fiber containing 1.2 mg valsartan. All measurements were done in triplicates. The fibers were placed into 50 mL solution and 2 mL samples were taken intermittently after 2, 5, 10, 15, 30, 60, and 90 min. The concentration of valsartan in the samples was determined by UV-Vis spectroscopy after calibration. Separate calibration curves were constructed for each of the four release media. After the determination of UV absorbance, the samples were replaced into the beaker containing the fibers. The experiments were carried out at room temperature without stirring.

### 2.5. Characterization

The encapsulation of the drug into the fibers was checked by Fourier transform infrared spectroscopy (FTIR). Spectra were recorded using a Bruker Tensor 27A (Bruker Corp., Billerica, MA, USA) apparatus in the wavelength range of 4000–400 cm^−1^ at 2 cm^−1^ resolution with 64 scans. Valsartan and cut fibers (2 mg) were mixed with KBR to prepare pastilles for the recording of the spectra. The spectra were evaluated using the Opus 2015 software. The amount of dissolved valsartan was determined by UV-Vis spectroscopy using a Unicam UV 500 type spectrophotometer (Unicam Ltd., Cambridge, UK) after calibration. The measurement was done in the wavelength range of 200–300 nm with 1 nm resolution and a scan rate of 120 mm/min.

The crystalline structure of the components was studied by differential scanning calorimetry (DSC) and X-ray diffraction measurements (XRD). DSC measurements were done on 3–5 mg fibers or on powder samples of the drug using a Perkin Elmer DSC IC apparatus (Perkin Elmer Inc., Waltham, MA, USA); samples were heated from 30–200 °C at the heating rate of 10 °C/min under N_2_ purge, cooled down at the same rate and then heated again. XRD patterns were recorded using a Philips PW 1830 diffractometer (Philips N.V., Amsterdam, The Netherlands). Measurements were carried out in the range of 2θ angles of 5–40°, with 0.04° increments and 1 s/step rate at the accelerating voltage of 40 kV and exciting current of 35 mA.

The morphology of the fibers was studied by digital optical (DOM) and scanning electron (SEM) microscopy. DOM micrographs were recorded using a Keyence VHX 5000 microscope (Keyence Corporation, Osaka, Japan) and they were used for the optimization of the electrospinning process. SEM micrographs were taken by using a JEOL JSM 6380LA (Jeol, Tokyo, Japan) scanning electron microscope (SEM) at the accelerating voltage of 15 and 25 kV. The micrographs were evaluated by the Image Pro Plus 7 (Media Cybernetics Inc., Rockville, MD, USA) image analysis software to determine the diameter of the fibers. The diameter of at least 100 fibers were measured for each composition.

## 3. Results and Discussion

The results are reported in several sections. The outcome of the optimization of the fiber spinning technology and the final spinning parameters are shown in the first section and then fiber characteristics as well as drug morphology and location are presented in the next two. The effect of polymer type and pH on drug release is analyzed in the next section, while release rate and the possible mechanism of drug release are discussed in the final section with possible consequences for practice.

### 3.1. Fiber Spinning, Parameters

The quality of electrospun fibers depends on many factors. The formation of the Taylor cone and successful spinning are determined by the characteristics of the solution (viscosity, polarity, surface tension), voltage, feeding rate, and on the evaporation of the solvent, which depends on the distance of the collector and environmental conditions like temperature and humidity. The viscosity of the solution is determined by the molecular weight of the polymer used and concentration. During the optimization of spinning technology we varied the polymer concentration, voltage, feeding rate, and collector distance.

**Figure 1:**
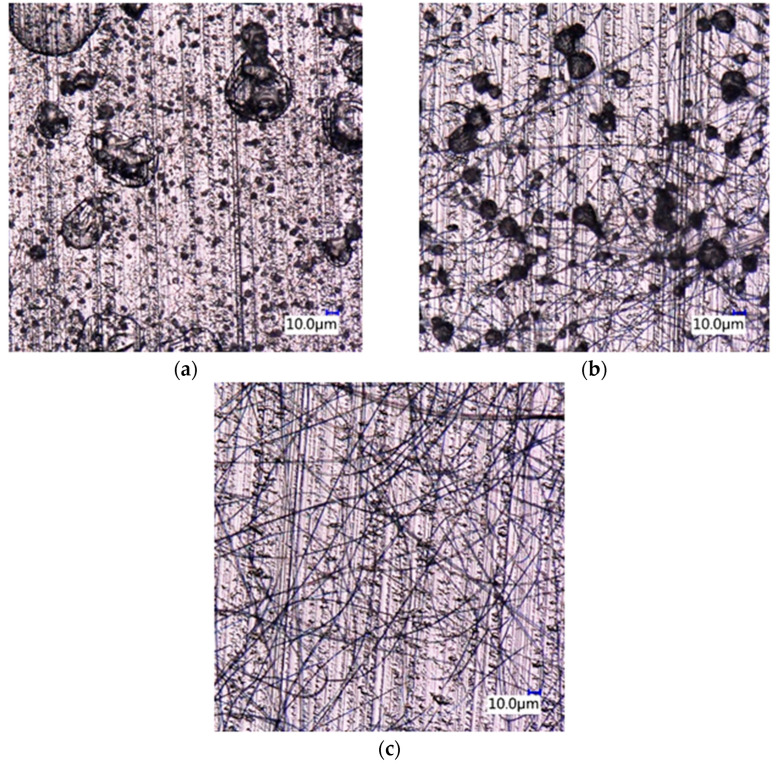
Effect of the concentration of PVP spinning solutions on the quality of the product; (**a**) 25, (**b**) 30, (**c**) 40 wt%.

Under certain conditions fibers did not form at all. The optimization process is demonstrated in the example of PVP in Figure 1. The series of micrographs shows the product of the spinning of PVP solutions at various concentrations. As Figure 1a shows fibers do not form at all at the concentration of 25 wt%, the solvent is deposited on the collector in the form of droplets. Besides droplets, also some fibers appear at the concentration of 30 wt%, but the quality of the product is still not sufficient at this polymer content (Figure 1b). Almost flawless, perfect fibers could be obtained at 40 wt% PVP concentration (Figure 1c) and at the same setting of the other parameters of the technology as before.

All the parameters mentioned in the first paragraph have been optimized in a similar way. Solution concentration proved to be the most sensitive parameter in the process, which influenced the quality of the fibers and the spinning process very strongly. The final parameters under which the fibers used in the further part of the study were produced are collected in Table 1. All three sets of parameters resulted in the production of fibers with acceptable quality which suited the purpose and could be used in the release study.

### 3.2. Fiber Characteristics

**Figure 2:**
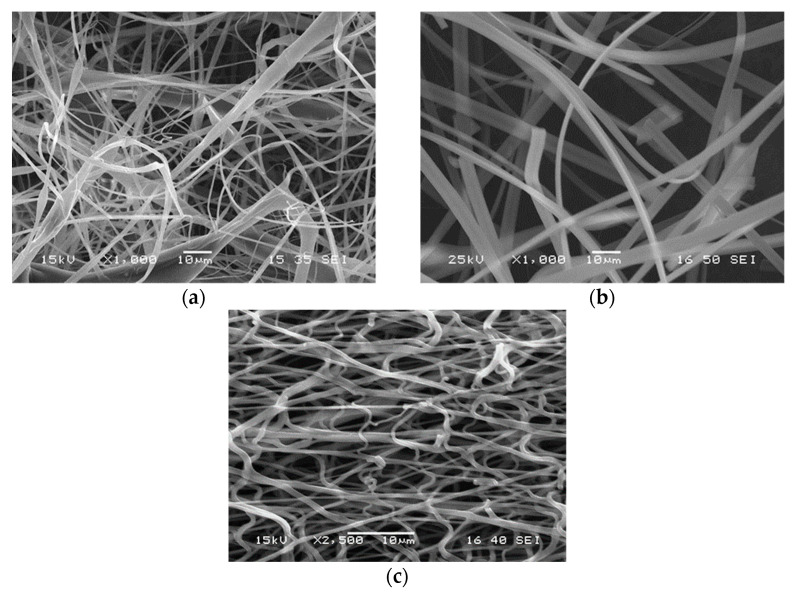
SEM micrographs recorded on electrospun fibers containing 20 wt% valsartan in various matrix polymers; (**a**) HPMC, (**b**) PVP, (**c**) PVA.

The technological parameters used for fiber spinning determine the shape, surface quality, and diameter of the fibers. The thickness of the fibers is especially important in the application in question, because it determines specific surface area and thus influences release rate. The morphology of the fibers spun under the conditions listed in Table 1 is presented in Figure 2. According to the SEM micrographs shown, fibers were obtained in all three cases indeed, but their shape and thickness was different for the three polymers. Thinner and thicker fibers were obtained from HPMC, the distribution of fiber diameter is relatively wide (Figure 2a). Fibers with more uniform thickness and smoother surface could be obtained from PVP (Figure 2b); the thickness of the fibers is in the same range as that of the HPMC fibers (see scale). The fibers produced from PVA are much thinner than in the other two cases and they seem to be more entangled, occasionally maybe even fused together (Figure 2c). Both the smaller thickness of the fibers and fusion indicate slower evaporation in spite of the larger distance to the collector (see Table 1), which might be the result of stronger interactions in the PVA solution.

**Figure 3:**
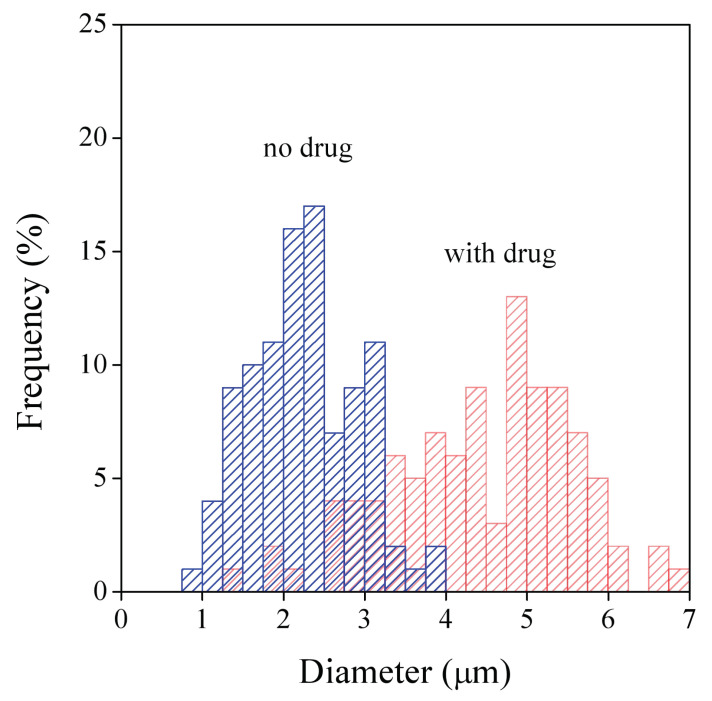
Distribution of the diameter of electrospun fibers prepared from PVP with and without the drug.

As mentioned above, the thickness of the fibers have large practical importance in drug release. The diameter of the fibers was determined by image analysis for both the neat fibers and for those containing the drug. The presence of the drug influenced fiber diameter only slightly in the HPMC and PVA matrices, but thickness increased considerably in the PVP matrix as a result of the incorporation of the drug. The distribution of fiber diameter is presented in Figure 3 for the PVP fibers with and without drug. The large difference in fiber diameters is clearly shown by the figure; thickness is almost twice as large in the presence of the drug than without it. Fiber diameters and the width of the distributions are collected in Table 2. The similarity of diameters with and without the drug for the HPMC and PVA matrices is clearly shown by the results, as well as the effect of the drug in the PVP matrix. PVA fibers are much thinner than the other two, as mentioned above, and the width of diameter distribution is also much smaller, the fibers are more uniform. Uniform fiber diameter is quite advantageous for drug release, it facilitates the control and the prediction of release.

### 3.3. Drug Morphology, Location

The location of the drug within the fibers is not trivial in the case when electrospun fibers are used as carrier matrix. Depending on the mutual solubility of the components, including the polymer, the solvents used, and the drug; this latter can be located within or among the fibers [41,42]. Moreover, the drug within the fiber can be crystalline or amorphous, precipitated as a separate phase or distributed as dissolved molecules. The location and the morphology of the drug in the device produced by fiber spinning influence drug release considerably.

**Figure 4:**
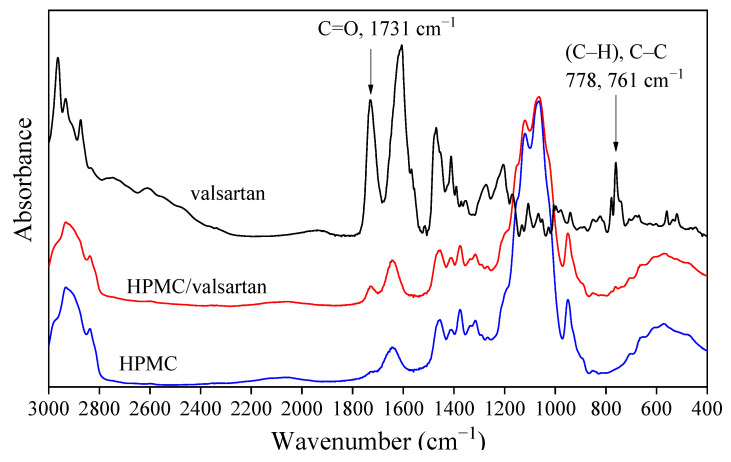
FTIR spectra recorded on valsartan, HPMC and electrospun HPMC fibers containing the drug at 20 wt%.

The incorporation of the drug into the fibers was checked by FTIR spectroscopy. The spectra recorded on the drug, on HPMC, and the polymer containing the drug are presented in Figure 4 as an example. The spectra considerably overlap with each other, but characteristic peaks can be determined for valsartan, mainly the intensive carbonyl group at 1731 cm^−1^ and the C–H bending and C–C bending vibrations at 778 and 761 cm^−1^. These characteristic peaks of the drug can be detected unambiguously in the spectra of the fiber containing the drug. Since drug particles could not be detected among the fibers in any of the SEM micrographs (see Figure 2), we must conclude that the drug is located within the fibers. Similar conclusions could be drawn from the FTIR study of the fibers prepared from the other two polymers. Spectroscopy unambiguously proved that valsartan was successfully incorporated into the fiber mats prepared from all three polymers and SEM indicated additionally that the drug is located within and not among the fibers.

**Figure 5:**
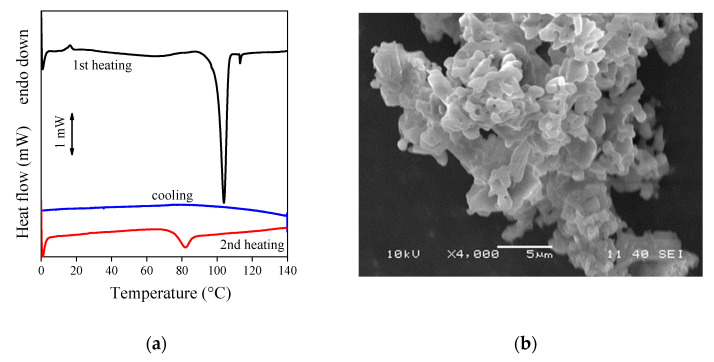
Morphology and thermal behavior of neat valsartan; (**a**) DSC traces recorded on the drug, (**b**) SEM micrograph of the neat drug powder.

Valsartan is a crystalline substance. However, its melting point was shown to vary between 50 and 110 °C indicating the existence of various crystal forms and/or varying crystallinity. Tran et al. [44] found, for example, that the type and crystallinity of valsartan depended also on the solvent used for crystallization that influenced drug release considerably. The DSC traces recorded on the drug used in our experiments are shown in Figure 5a. The curve recorded in the first heating exhibits a sharp melting peak at around 105 °C indicating large crystallinity and crystals with considerable regularity. Crystalline structure is also confirmed by the SEM micrograph recorded on the drug (Figure 5b). On the other hand, the endothermic peak of crystallization could not be detected during the cooling run, and both melting temperature and crystallinity were much smaller in the second heating run. These results clearly indicate that the crystal form and crystallinity of valsartan are strongly influenced by the conditions of crystallization indeed, and thus the process and technology of fiber spinning must also change the structure of the drug considerably.

**Figure 6:**
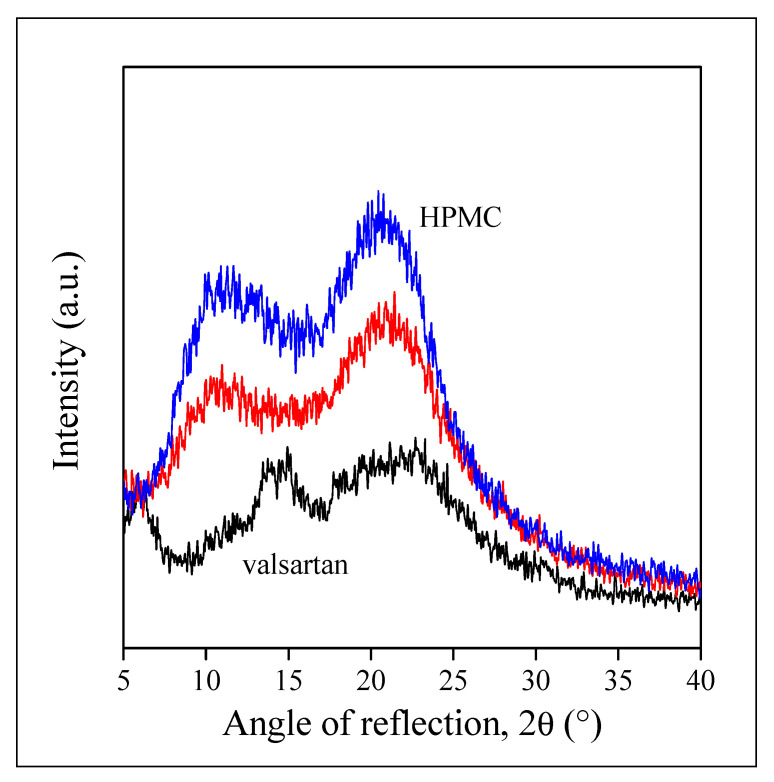
XRD patterns recorded on valsartan, HPMC, and HPMC fibers containing the drug at 20 wt%.

X-ray diffraction was used to check the form of the drug within the fibers and the possible crystallinity of the polymer. The patterns recorded on valsartan, the neat HPMC fibers, and on fibers containing the drug are presented in Figure 6. The pattern of valsartan shows its crystallinity, but also that it is not completely crystalline and the crystals are not very regular, which partly contradicts the conclusion drawn from the DSC analysis. HPMC and the fibers containing the drug are completely amorphous, no trace of the crystalline reflection of valsartan can be detected on the pattern. Based on these results we can conclude that valsartan is dispersed within the polymer fibers in amorphous form that is expected to facilitate drug release.

### 3.4. Drug Release

**Figure 7:**
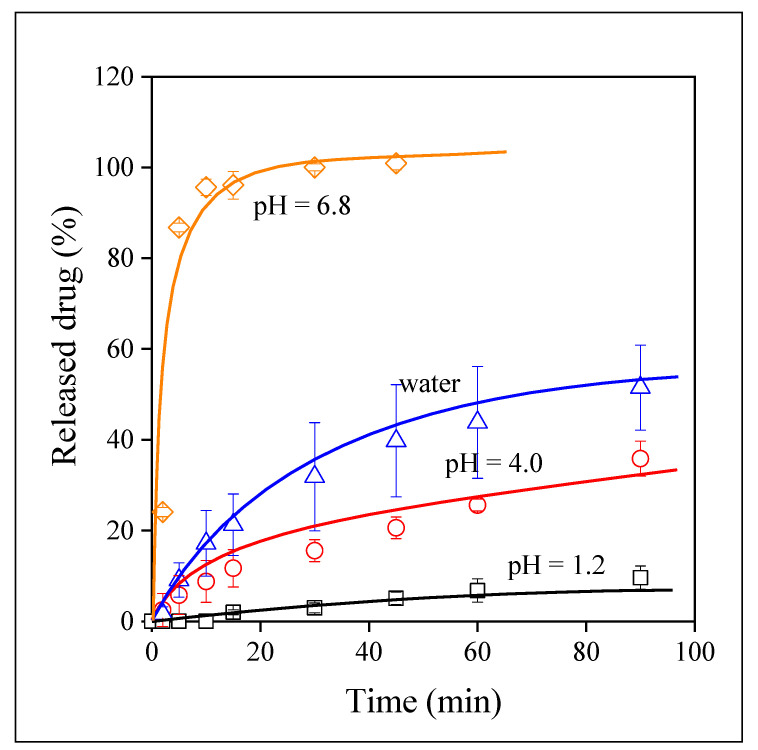
Time dependence of the dissolution of valsartan in various media. Symbols: (☐) pH 1.2, (○) pH 4.0, (◇) pH 6.8, (△) water.

Valsartan is a BSC II type drug with limited solubility in water-based media. Moreover, it is slightly acidic thus its solubility and dissolution depend very much on the pH of the solution. Accordingly, the dissolution characteristics of valsartan as well as the release of the drug from electrospun fibers were studied at three different pH values and also in distilled water. The dissolution of the neat drug in the four media is presented in Figure 7. The strong dependence of dissolution on pH is amply demonstrated by the figure. Dissolution is quite fast and complete at pH 6.8, but very slow and extremely limited at pH 1.2. It is interesting to note that dissolution in distilled water is much slower than at pH 6.8 and solubility is also much smaller, about 50 % under the conditions of the experiments. Since the pH of distilled water is very close to 7.0, the phenomenon is difficult to understand. We must assume that the ionic moieties of the dissolution media influence and possibly facilitate the dissolution of the drug. Since the pH of the digestive tract is strongly acidic, the solubility of valsartan must be improved considerably.

**Figure 8:**
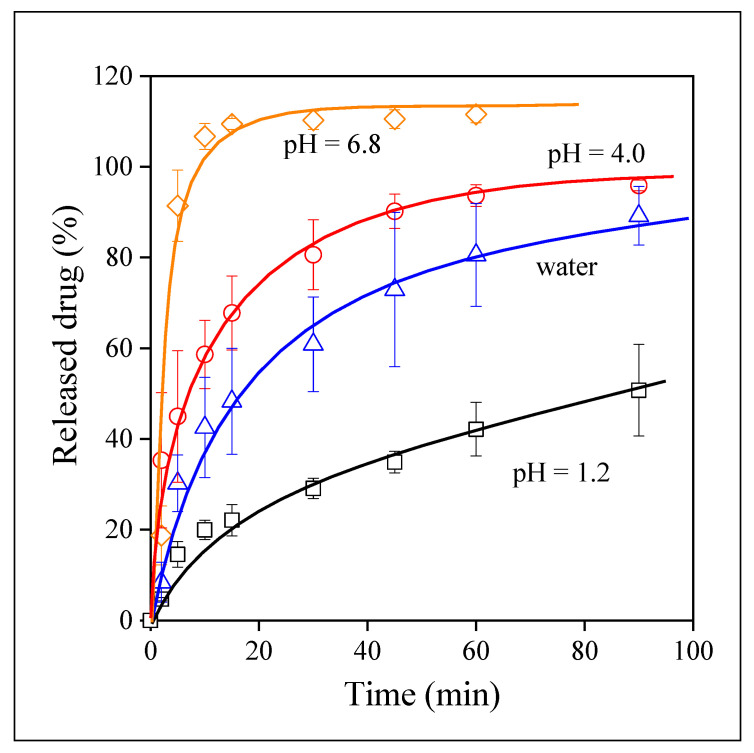
Release kinetics of valsartan from PVP electrospun fibers. Symbols: (☐) pH 1.2, (○) pH 4.0, (◇) pH 6.8, (△) water.

The release of the drug from the electrospun fibers was studied under the same conditions. The results obtained on the PVP fibers are presented in Figure 8. Solubility definitely increased at all pH values and also in distilled water, i.e., the incorporation of the drug into this water-soluble polymer is beneficial and we can hope that bioavailability also increases. The pH of the solution has practically the same effect on solubility as before, but at a higher level. The smallest solubility is measured at pH 1.2, the final value is close to 40%, and solubility reaches values of around 90% at pH 4.0 and in distilled water. Improved solubility is obviously the result of the amorphous nature, and better distribution of the drug as well as the larger specific surface area offered by the fibers, i.e., larger contact surface. The release of the drug from the HPMC fibers is very similar to that observed in PVP, but naturally the values are somewhat different; the improvement in solubility at pH 4.0 and in water is smaller and pH sensitivity is weaker than in the PVP fibers.

**Figure 9:**
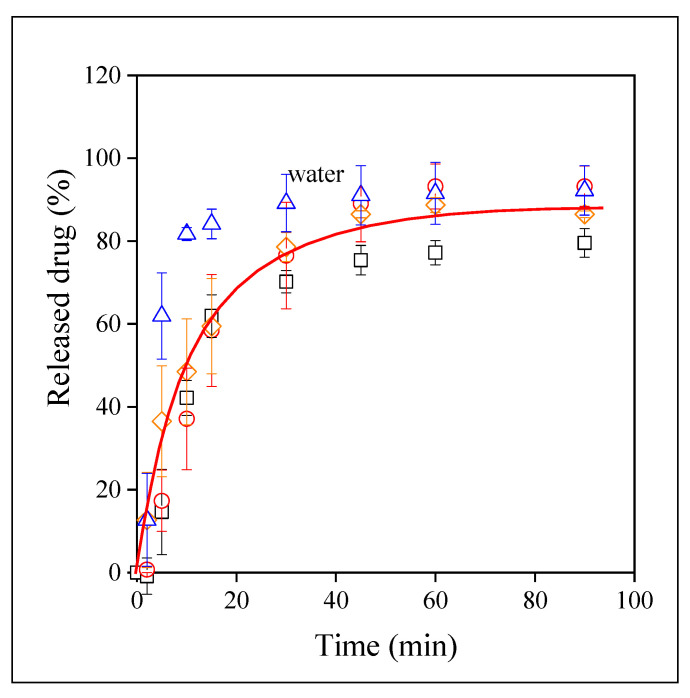
The kinetics of drug release from electrospun fibers prepared from PVA. Symbols: (☐) pH 1.2, (○) pH 4.0, (◇) pH 6.8, (△) water.

The behavior of the drug incorporated into the PVA matrix is very interesting. The time dependence of release from these fibers is shown in Figure 9. The largest extent of release is achieved in water that is quite surprising. Moreover, the pH dependence of release is practically absent in this case, the rate of release is similar at all three pH values and the same extent of solubility, around 80–85% is reached in all cases. Although the release of the drug from PVA is not complete, the improvement is large, and the independence of release of pH compensates for the slight decrease of equilibrium solubility.

**Figure 10:**
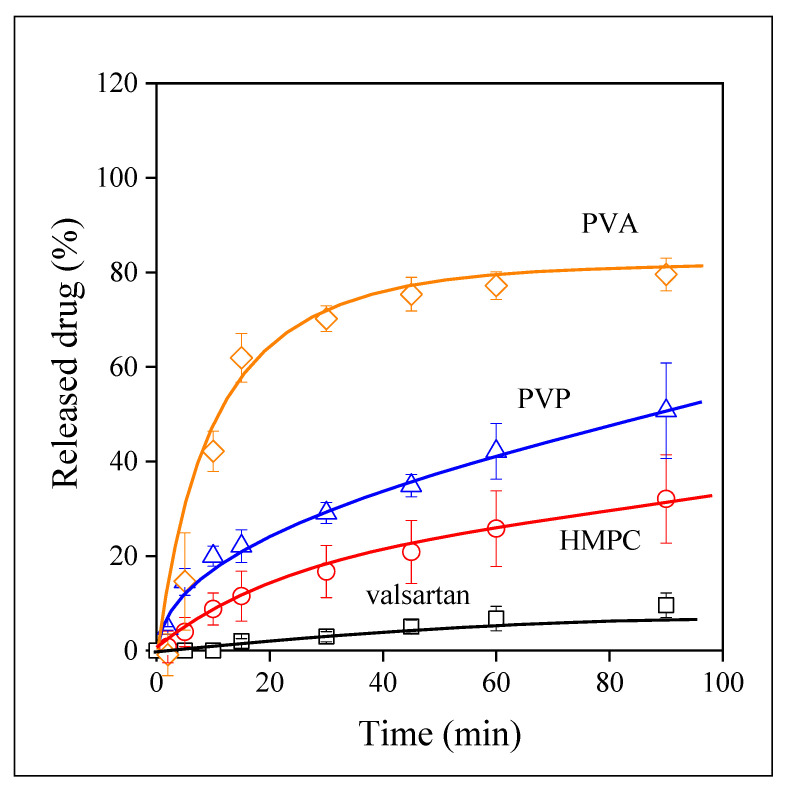
Comparison of the dissolution of valsartan and the kinetics of its release from the electrospun fibers produced from various water-soluble polymers at pH 1.2. Symbols: (☐) valsartan, (○) HPMC, (◇) PVA, (△) PVP.

The improvement of solubility of the drug as a result of its incorporation into polymer matrices is demonstrated well by Figure 10. The dissolution of the drug and its release from the three polymers is compared at pH 1.2, because of the importance of acidic conditions due to the high acidity of the stomach. Solubility improves in the same degree when the HPMC and PVP matrices are used, as mentioned above, but very large solubility is achieved in PVA. The rate of release also increases considerably in this latter matrix. The results presented in Figure 10 indicate the effect of several factors. The form of the drug, its distribution, the surface area are factors acting in all matrices. However, some other factor must also play a role in the PVA matrix which increases the rate of release and improves solubility. Further study and considerations are needed to reveal this factor and to fully utilize its potentials.

### 3.5. Release Rates, Mechanisms

Although the visual observation of the correlations presented in Figure 7, Figure 8, Figure 9 and Figure 10 offers valuable information about the release of valsartan from the electrospun polymeric fibers and about the factors influencing it, the quantitative evaluation of the results may allow a deeper insight into the process. The kinetics of dissolution can be described by the Noyes–Whitney equation [45]:

(1) $$\frac{dc\left(t\right)}{dt}=\frac{A\;D}{V\;h}\;\left[c_{s}-c\left(t\right)\right]$$

where *c* is the concentration of the drug, *t* time, *A* surface area, *D* diffusion coefficient, *V* the volume of the dissolution medium, *h* is the thickness of the diffusion layer, and *c_s_* solubility. The integration of the equation and rearrangement leads to

(2) $$c\left(t\right)=c_{s}\;\left(1-e^{-kt}\right)$$

where *k* contains the constants of Equation (1), i.e., *k* *= AD/Vh* and corresponds to the overall rate of dissolution. Equation (2) was fitted to all dissolution correlations to determine solubility (*c_s_*) and the rate of dissolution.

**Figure 11:**
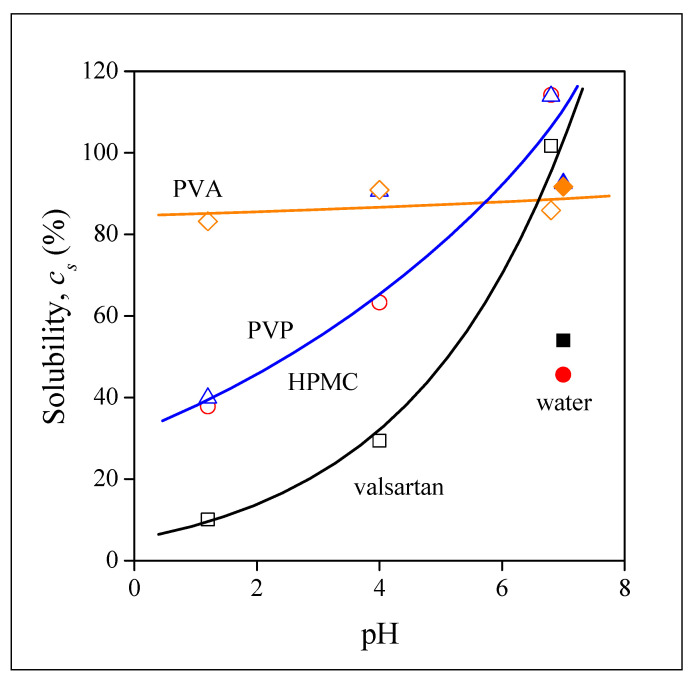
pH dependence of the equilibrium dissolution of neat valsartan and that of valsartan released from electrospun fibers prepared from different polymers. Symbols: (☐) neat valsartan, (○) HPMC, (◇) PVA, (△) PVP; full symbols indicate dissolution into distilled water.

Equilibrium solubility is plotted against the pH of the dissolution medium in Figure 11. The results of the figure confirm the observation done earlier. Complete solubility is reached for valsartan and for the drug released from the HPMC and PVP matrices at pH 6.8, but it is very small at pH 1.2 and the dependence of solubility on pH is very strong for these materials. The dissolution of the drug from the PVA matrix is large and completely independent of pH. The dissimilar behavior indicates different mechanisms for drug release. The similarity of the behavior of neat valsartan and that of the electrospun fibers containing the drug indicates that in these cases release is dominated by the dissolution of the drug. Accordingly, the dissolution of the polymers must be faster than that of the drug itself, the characteristics of the drug dominate and thus solubility and its dependence on pH are practically the same. The differences in the values are caused by the form of the drug, homogeneity, and surface area. In the case of the PVA fibers, on the other hand, the dissolution of the polymer must be slower and thus it is the dominating factor leading to a larger and pH independent release of valsartan. The tentative explanation presented here is plausible, but needs verification in the future. Finally, we must call attention here to the solubility values measured in water. The points are indicated by full symbols. With the exception of PVA, solubility is always smaller in water than in the buffers, which indicates the role of ionic species in the dissolution of valsartan.

**Figure 12:**
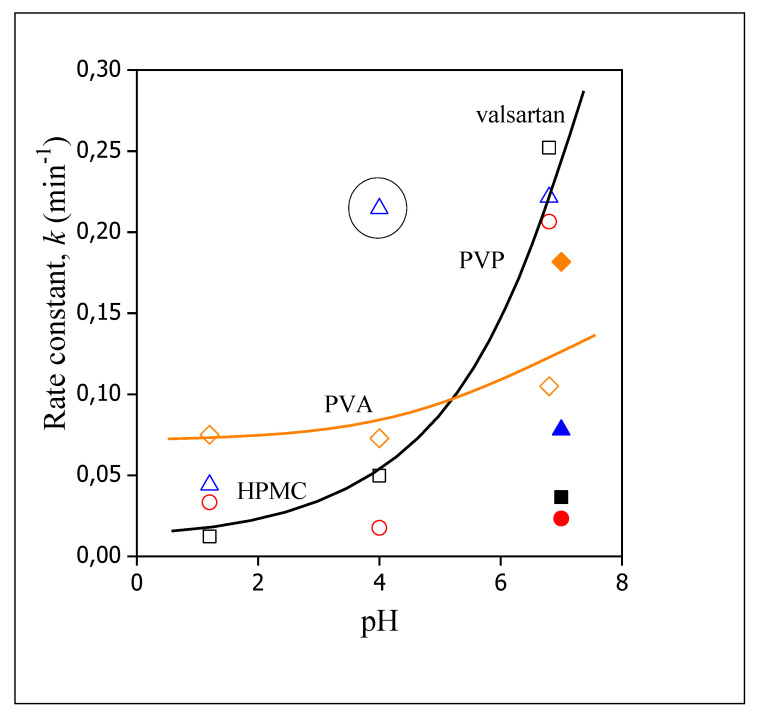
Effect of the pH of the dissolution medium on the rate of dissolution of valsartan and the release of the drug from electrospun fibers. Symbols: (☐) neat valsartan, (○) HPMC, (◇) PVA, (△) PVP; full symbols indicate dissolution into distilled water.

The rate of dissolution is plotted against pH in Figure 12. The correlations are somewhat more difficult to interpret in this case. The rates determined for the neat valsartan and for the fibers prepared from HPMC and PVP are similar which confirms our conclusion about the determining role of valsartan dissolution. The value obtained in the PVP matrix at pH 4.0 is much larger (indicated by a circle), but it is a single, deviating point, which needs verification. The large deviation might be a real effect, but also might result from an error in the measurements or fitting. The different behavior of the device prepared from PVA is evident also in this figure. Rates are commensurable at smaller pH values to those obtained for the other fibers, but depend only very slightly on pH. According to the results of the release study and modelling, the most advantageous matrix for the preparation of devices offering larger bioavailability of valsartan is PVA. The results also indicate that the drug can be administered orally, since all components are approved materials and the fast dissolution of the carrier polymer as well as that of the encapsulated valsartan would allow oral administration, and increase bioavailability considerably.

## 4. Conclusions

In an attempt to improve the solubility of valsartan, a BCS II drug, electrospun fibers containing the drug were prepared from three water-soluble polymers, HPMC, PVP, and PVA. The drug was homogenously distributed within the fibers in the amorphous form. The presence of the drug interfered with the spinning process only slightly; the diameter of the fibers were in the same range as without the drug for the HPMC and the PVA fibers, while it doubled in PVP. The incorporation of the drug into the fibers increased its solubility in all cases compared to that of the neat drug. The solubility of the drug itself depends very much on pH and this sensitivity remained the same in the HPMC and PVP fibers; the release of the drug was dominated by the dissolution behavior of valsartan itself. On the other hand, solubility and the rate of release were practically independent of pH in the PVA fibers. The different behavior is explained by the dissolution rate of the respective polymer, which is larger for HPMC and PVP, and smaller for PVA than the dissolution rate of the drug. The larger extent of release can be explained by the lack of crystallinity of the drug, its better dispersion, and larger surface area of the fibers. Considering all facts, the preparation of electrospun devices from valsartan and water-soluble polymers is beneficial, and the use of PVA is more advantageous than that of the other two polymers.

## Figures and Tables

**Table 1:** Optimized technological parameters used for the electrospinning of fibers prepared from the polymers used in this study.

Polymer	Concentration (wt%)	Voltage (kV)	Feeding Rate (mL/h)	Collector Distance (mm)
HPMC	10	20	5	100
PVP	40	20	7	125
PVA ^a)^	15	15	0.8	140

^a)^ water was replaced by 30 vol% ethanol in the solution in order to improve the efficiency of spinning and fiber quality.

**Table 2:** Diameter of the fibers spun from the polymers used in the study and the effect of the incorporation of the drug into them.

Polymer	Average Diameter (µm)	
	No Drug	With Drug
HPMC	2.0 ± 1.0	1.5 ± 0.7
PVP	2.3 ± 0.7	4.4 ± 1.1
PVA	0.5 ± 0.1	0.7 ± 0.2

## Data Availability

Not applicable.
